# Therapeutic Potential of β-Caryophyllene: A Dietary Cannabinoid in Diabetes and Associated Complications

**DOI:** 10.3390/nu12102963

**Published:** 2020-09-28

**Authors:** Hebaallah Mamdouh Hashiesh, M.F. Nagoor Meeran, Charu Sharma, Bassem Sadek, Juma Al Kaabi, Shreesh K. Ojha

**Affiliations:** 1 Department of Pharmacology & Therapeutics, College of Medicine and Health Sciences, United Arab Emirates University, Al Ain P.O. Box 17666, UAE; 201990080@uaeu.ac.ae (H.M.H.); nagoormeeran1985@uaeu.ac.ae (M.F.N.M.); bassem.sadek@uaeu.ac.ae (B.S.); 2 Department of Internal Medicine, College of Medicine and Health Sciences, United Arab Emirates University, Al Ain P.O. Box 17666, UAE; charusharma@uaeu.ac.ae (C.S.); j.kaabi@uaeu.ac.ae (J.A.K.); 3 Zayed Center for Health Sciences, United Arab Emirates University, Al Ain P.O. Box 17666, UAE

**Keywords:** β-caryophyllene, diabetes, essential oils, hyperglycemia, insulin resistance, inflammation, natural products, oxidative stress, sesquiterpenes

## Abstract

Diabetes mellitus (DM), a metabolic disorder is one of the most prevalent chronic diseases worldwide across developed as well as developing nations. Hyperglycemia is the core feature of the type 1 diabetes mellitus (T1DM) and type 2 diabetes mellitus (T2DM), following insulin deficiency and impaired insulin secretion or sensitivity leads insulin resistance (IR), respectively. Genetic and environmental factors attributed to the pathogenesis of DM and various therapeutic strategies are available for the prevention and treatment of T2DM. Among the numerous therapeutic approaches, the health effects of dietary/nutraceutical approach due to the presence of bioactive constituents, popularly termed phytochemicals are receiving special interest for pharmacological effects and therapeutic benefits. The phytochemicals classes, in particular sesquiterpenes received attention because of potent antioxidant, anti-inflammatory, and antihyperglycemic effects and health benefits mediating modulation of enzymes, receptors, and signaling pathways deranged in DM and its complications. One of the terpene compounds, β-caryophyllene (BCP), received enormous attention because of its abundant occurrence, non-psychoactive nature, and dietary availability through consumption of edible plants including spices. BCP exhibit selective full agonism on cannabinoid receptor type 2 (CB2R), an important component of endocannabinoid system, and plays a role in glucose and lipid metabolism and represents the newest drug target for chronic inflammatory diseases. BCP also showed agonist action on peroxisome proliferated activated receptor subtypes, PPAR-α and PPAR-γ, the main target of currently used fibrates and imidazolidinones for dyslipidemia and IR, respectively. Many studies demonstrated its antioxidant, anti-inflammatory, organoprotective, and antihyperglycemic properties. In the present review, the plausible therapeutic potential of BCP in diabetes and associated complications has been comprehensively elaborated based on experimental and a few clinical studies available. Further, the pharmacological and molecular mechanisms of BCP in diabetes and its complications have been represented using synoptic tables and schemes. Given the safe status, abundant natural occurrence, oral bioavailability, dietary use and pleiotropic properties modulating receptors and enzymes, BCP appears as a promising molecule for diabetes and its complications.

## 1. Introduction

Diabetes mellitus (DM) is the most prevalent metabolic disorder and continuing to affect a large number of people worldwide. The International Diabetic Federation reported that the prevalence of diabetes is increasing worldwide to epidemic proportions; in 2017, 425 million people were affected by DM and the number is expected to rise to 629 million in 2045 [1]. DM mainly involves perturbation of metabolic pathways that influence carbohydrate and lipid metabolism; the two major cellular substrates result in the generation of injurious metabolites [2]. The uncontrolled DM gives rise to microvascular and macrovascular complications affecting structural and functional changes in tissues and may result in organ dysfunction or failure [3]. The exact underlying molecular mechanisms of the pathogenesis of DM are not fully recognized [4]. However, the pathogenesis mainly involves beta-cell dysfunction, reduced amount or sensitivity of insulin or insulin resistance (IR) along with oxidative stress, inflammation, mitochondrial dysfunction, and apoptosis [2,4].

Glycemic control is one of the main aims of treatment in the management of DM and to delay the onset and progression of diabetic complications [5,6]. Though, the currently available therapeutic strategies are effective but adverse effects, inadequate effectiveness in metabolic and glucoregulatory mechanisms necessitate exploring novel therapeutic and preventive agents for the prevention of diseases related to DM in particularly type 2 diabetes mellitus (T2DM) and IR [5]. Thus, there is the need for more safer and effective drugs and therapeutic strategies to prevent various short- and long-term risk factors associated with diabetes [7,8]. The risk factors, genetic and environmental including obesity, sedentary lifestyle, smoking, alcohol consumption, and diet have been considered to be a major reason of the alarming rise in T2DM worldwide and are the focus of therapeutic targeting to alleviate impaired insulin secretion and IR [9,10]. In environmental factors, diets have received interest in the physiological and pharmacological perspective based on the data from epidemiological, preclinical studies and few clinical studies demonstrated that nutraceuticals, functional foods, dietary/food supplements play role in improving postprandial hyperglycemia and adipose tissue metabolism thus modulating glucose and lipid metabolism [11,12,13].

Mounting evidence indicate that various fruits, vegetables, and whole grains including fermented products, legumes, nuts, green tea, spices, propolis, olive oil, red wine (resveratrol), herbs, and spices are beneficial in T2DM, obesity, and metabolic syndrome due to the presence of non-nutritive secondary metabolites termed as phytochemicals [14,15,16,17,18]. The dietary bioactive phytochemicals play significant role in maintaining health and wellness attributed to their potent pharmacological effects that translate to medicinal benefits [19]. Phytochemicals belong to numerous classes such as polyphenols, terpenoids, flavonoids, alkaloids, sterols, pigments, and unsaturated fatty acids [20]. Many of the edible plants and phytochemicals are suggested to be useful in T2DM and delay and or prevent complications exerting a pronounced beneficial effect in controlling hyperglycemia due to potent antioxidant and anti-inflammatory effects [14,15,16,17].

Among numerous phytochemical classes, specifically, terpenes commonly consumed in plants including edible and medicinal garnered attention because of their potent bioactivities, safety, druggable properties, and potential to target enzymes and receptors that influence glucose and lipid metabolism [18]. In sesquiterpene compounds, β-caryophyllene (BCP), a bicyclic, non-psychoactive compound of wide natural occurrence in essential oils of many edible plant including spices like clove, cinnamon, thyme, oregano, and black pepper received interest because of its potent pharmacological properties and therapeutic benefits [21,22,23]. BCP naturally occurs in plants with its isomers such as (*Z*)-β-BCP also known as isocaryophyllene; a *cis*-double bond isomer or α-humulene, also known as *α*-caryophyllene, a ring-opened isomer or in a mixture with *β*-caryophyllene oxide, an oxidation product of BCP. The cyclobutene ring in the chemical structure of BCP imparts chemical stability and high lipophilicity [23]. It received approval by European agencies, Flavor, and Extract Manufacturers Association (USFDA), for safe use in foods as additive, preservative, and flavor enhancer. USFDA listed BCP “generally regarded as a safe (GRAS)” for use in food and cosmetics. Since its recognition as a dietary cannabinoid, BCP has received interest for studies to investigate its pharmacological properties and therapeutic potential [22]. Sharma et al. 2016 have reviewed numerous pharmacological properties of BCP including antioxidant, anti-inflammatory, antimicrobial, chemopreventive, nephroprotective, cardioprotective, neuroprotective, and nephroprotective along with pharmaceutical characteristics [23].

Gertsch and colleague for the first time demonstrated a selective and full agonist activity of BCP on cannabinoid type 2 receptors (CB2R), a component of the endocannabinoid system consisting of endocannabinoid ligands that exert their action by binding to two well-characterized cannabinoid receptors type 1 and type 2 (CB1R and CB2R) or endocannabinoid metabolizing enzymes, fatty acid amide hydrolase (FAAH), and monoacylglycerol lipase (MAGL) [22]. BCP showed binding affinity to the CB2R in nanomolar concentrations and exhibited potent cannabimimetic actions [22]. BCP does not bind to the centrally expressed CB1 receptors, thus does not exert psychomimetic effects. The activity of CB1 or CB2R can be modulated directly by ligand binding or indirectly by the modulation of the level of endocannabinoids. The role of cannabinoids has been shown to suppress inflammation and oxidative stress, the two-common accompaniment of DM by targeting CB2R [24].

Recently, numerous experimental studies revealed that activation of CB2R by agonists have significant pharmacological effects such as anti-inflammatory, immunomodulatory, antioxidant, cardioprotective, hepatoprotective, gastroprotective, neuroprotective, nephroprotective, and atheroprotective and found to be beneficial in controlling hyperglycemia, IR, and dyslipidemia [25,26]. In the past few years, significant attention is being focused on the possibility of developing novel drugs that can modulate the endocannabinoid system, in particular, activate CB2R, knowing the adverse psychiatric effects of CB1 receptor modulation and in particular, more emphasis has been given to the CB2R targeting cannabinoids of natural origin over the synthetic ones [24,26,27]. BCP is devoid of psychoactivity, a common feature of many cannabinoids because of the modulation of CB1 receptors. CB2R selectivity and affinity makes BCP a promising candidate for drug development targeting the endocannabinoid system, a relatively new therapeutic target.

BCP is a molecule of special interest with high presence in cannabis as well as the non-cannabis plants including more than 2000 plants and termed as a dietary cannabinoid [22,23]. BCP has been suggested to be used as a nutraceutical, functional food, and dietary supplement because of its dietary bioavailability, ample natural occurrence, and multiple therapeutic benefits [23]. Many formulations such as suspension, PEG formulations, liposomes, wound dressings, hydrogels, and nanoemulsions for topical use, inclusion complexes with cyclodextrins, as well as nanocomposites and nanoparticles have been developed [28]. These formulations have been shown to overcome the physicochemical issues such as lipophilicity, low stability, and bioavailability that may hinder the pharmaceutical development.

In the past few years, the number of studies demonstrated that BCP possesses a wide range of pharmacological properties including anti-inflammatory and antioxidant and the potential to correct hyperglycemia and improve insulin secretion and sensitivity, thus appearing as a promising molecule for diabetes and its complications [29]. Given the role of oxidative stress and low-grade chronic inflammation in the pathogenesis of DM [30], the role of cannabinoid ligands in DM and associated complications have been demonstrated in numerous studies [31].

Convincing number of preclinical studies and few clinical studies demonstrated its pharmacological effects and molecular mechanisms, redox and immune-inflammatory axis, and its beneficial effects in low grade chronic inflammatory diseases including DM and its complications [21,22,23]. The current review represents a comprehensive account of preclinical and a few clinical studies concerning the effects of BCP in diabetes and associated diabetic complications. The review also delineates the pharmacological and molecular mechanisms of BCP in diabetes and its complications. The pharmacological effects and mechanisms are summarized in synoptic tables and schemes.

## 2. Effects and Possible Mechanisms of BCP on Diabetes

### 2.1. Effect on Pancreatic Islet β-Cells and Insulin Secretion

#### 2.1.1. In Vitro Studies Showing Effects of β-Caryophyllene

Pancreatic β-cells releases insulin to control the sugar levels in the blood when blood glucose concentration increases. BCP has been shown to influence dysfunctional pancreatic β-cells and insulin secretion. Suijun, et al. [32] have demonstrated the roles of BCP on glucose-stimulated insulin secretion (GSIS) and underlying mechanisms. GSIS is vital for the regulation of metabolic fuel homeostasis and its deregulation, and is a crucial component in the failure of β-cells in T2DM. BCP treatment to MIN6 β-cells dose-dependently enhanced GSIS [32]. However, when MIN6 cells were treated with a CB2R blocker or specific RNA interference abrogated the positive effects of BCP on GSIS that demonstrated CB2R activation-dependent effect of BCP on GSIS and potential in T2DM.

The trafficking and fusion of insulin secretory vesicle to the plasma membrane are regulated by coordinated signaling events. Previous studies have demonstrated that small G-proteins e.g., ADP-ribosylation factor 6 (Arf6), cell division cycle 42 (Cdc42), and Ras-related C3 botulinum toxin substrate 1 (Rac1) play an important role in regulating signaling events involved in insulin trafficking [33]. It has been shown that Arf6 regulates insulin secretion induced by glucose, GTPCS, and membrane depolarization and as an upstream signaling factor of Rac1 and CDc42, play role in the regulation of GSIS [34]. Cdc42 is a crucial player in progression of DM by regulating insulin synthesis and mobilization of insulin granule and cell membrane exocytosis through activating a series of downstream factors [35]. The Rho family GTPase Rac1 has been shown to regulate insulin-stimulated GLUT4 translocation and glucose transport in cultured muscle cells [36]. Suijun, Zhen, Ying, and Yanfang [32] showed that treatment of MIN6 β-cells with BCP activated the expression of small G protein Arf6 as well as Rac1 and Cdc42. Importantly, silencing of Arf6 abolished the effects of BCP on GSIS that is suggestive of Arf6-mediated effects of BCP on GSIS. BCP was found to improve GSIS in pancreatic β-cells regulating Arf6, representing a unique mechanism in glucose homeostasis mediated by CB2R agonist.

In addition, Kumawat and Kaur [37] evaluated the insulinotropic effect of BCP and its combination with semi-essential amino acid L-arginine on insulin secretion in rat insulinoma (RIN-5F) cell lines following glucose challenge. The co-administration of BCP and L-arginine was found to significantly increase insulin secretion, and activate CB2R that is believed to promote the entry of calcium and enhance the insulin secretion from the pancreatic β-cells [26]. L-arginine was also found to maintain integrity and regeneration of pancreatic β-cells. The combination of BCP and L-arginine was found superior over BCP and L-arginine alone in restoring insulin secretion and pancreatic β-cells integrity in rat insulinoma cells. In addition, BCP was found to induce the expression of small G proteins, which regulates trafficking of insulin-laden secretory granules to plasma membranes mediating CB2R. The effect of BCP on insulin homeostasis specifically on insulin secretion, glucose uptake and translocation, and associated metabolic pathways are summarized in Table 1. The available studies were suggestive of the underlying mechanism of BCP as an insulin secretagogue as well as an insulin sensitizer in experimental studies.

#### 2.1.2. In Vivo Studies Showing Antihyperglycemic Effects of β-Caryophyllene

Basha and Sankaranarayanan [42] assessed the role of BCP on glucose homeostasis in streptozotocin (STZ)-induced DM in rats. Glucose homeostasis is ascribed to endogenous glucose production and its utilization by target tissues. Insulin controls the activities of key enzymes, so its insufficiency results in a deregulated carbohydrate metabolism [43]. Normally, insulin increases glucose utilization by enhancing the activities of essential enzymes such as hexokinase, pyruvate kinase, and glucose-6-phosphate dehydrogenase (G6PDH). Hexokinase, an insulin-dependent enzyme phosphorylates glucose to glucose-6-phosphate and plays a significant role in the maintenance of glucose homeostasis. Pyruvate kinase, ubiquitously expressed glycolytic enzyme, causes the conversion of phosphoenolpyruvate to pyruvate with ATP generation. G6PDH channelizes glucose via the pentose phosphate pathway [44,45]. BCP has been found to positively influence the activities of key enzymes that are significantly reduced under diabetic conditions and leads to the impaired glucose utilization in target tissues, Basha and Sankaranarayanan [42].

Basha and Sankaranarayanan [46] further determined the impact of BCP on different components of glycoproteins in STZ-induced T2DM rats. Glycoproteins (e.g., hexose, hexosamine and sialic acid) are the conjugated proteins that consist of one or more covalently linked carbohydrate chains which contribute to the extracellular matrix structure in animal cells [47]. Deranged hexose, hexosamine, fucose, and sialic acid metabolism has been reported in diabetic conditions. Many studies have demonstrated that variation in the components of glycoprotein are a consequence of deranged carbohydrate metabolism [48]. BCP upon oral treatment to STZ-induced diabetic rats significantly reduced blood glucose and increased plasma insulin to near normal level accompanied with a significant reduction of plasma levels of glycoproteins (hexose, hexosamine, fucose, and sialic acid). Additionally, sialic acid levels were reduced in the liver and kidney of diabetic rats with a concomitant rise in protein-bound hexose, hexosamine, and fucose. BCP treatment to diabetic rats significantly regresses these altered glycoprotein components to near normal in target tissues.

The effect of BCP on hyperglycemia-mediated oxidative stress and inflammation in STZ-induced DM in rats was evaluated. Oxidative stress is a common hallmark for the crucial pathways involved in the development and progression of DM [30]. Dysfunction of pancreatic β-cell results from deregulated metabolic factors, oxidative stress, and inflammatory responses [49]. Oxidative stress activates a series of stress/inflammatory signaling pathways that have a negative impact on insulin signaling [50]. Islets of infiltrating immune cells produce various inflammatory cytokines, which play a significant role in the destruction of β-cells in pancreatic tissue [51]. BCP supplementation significantly decreased blood glucose and improved serum insulin levels in STZ-induced diabetic rats. Furthermore, it remarkably augmented both enzymatic and non-enzymatic antioxidants and reduced rise in inflammatory cytokines (TNF-α and IL-6) in pancreas and plasma. BCP upon oral administration appears to alleviate hyperglycemia and protect β-cells by increasing insulin release and preventing oxidative stress and inflammatory cytokines in pancreas and plasma of diabetic rats [29].

A pivotal step in the pathogenesis of T2DM is the amplified hepatic gluconeogenesis, which is regulated mainly by gluconeogenic enzymes including G6pase, F1, 6Bpase, PEPCK, and PC. In diabetic rats, enhanced activities of gluconeogenic enzymes were detected in hepatic and peripheral tissues. In the diabetic conditions, insulin deficiency augments the activities of gluconeogenic enzymes that results in increased production of endogenous glucose [52]. Glycogen is the primary intracellular storage form of glucose in the liver and skeletal muscle. Insulin promotes deposition of intracellular glycogen through stimulating glycogen synthase (GS) and inhibiting glycogen phosphorylase (GP) which is the key regulatory enzymes to catalyze glycogen synthesis and catabolism, respectively. BCP upon oral treatment to diabetic rats mitigated the altered activities of carbohydrate metabolic enzymes (HK, PK, G6PDH, gluconeogenic enzymes, and GP and GS) to near normal levels along with reducing blood glucose and improving insulin levels in plasma. BCP also showed insulinotropic property in the immunohistochemical studies. BCP enhances secretion of insulin and restores glucose homeostasis via modulation of the activities of major enzymes involved in glucose utilization and production in target tissues.

In a previous preclinical study Kumawat and Kaur [37], evaluated the antidiabetic potential of BCP, L-arginine, and its combination in diabetic rats. The combination of BCP with L-arginine to STZ-induced diabetic rats significantly reduced glucose, demonstrating influence on insulin secretion, plays a significant role in the regulation of glucose levels in T2DM. These positive effects were attributed to the stimulation of CB2Rs [53] as well as pancreatic β-cell regeneration activity of L-arginine [54]. Furthermore, this combination resulted in normalization of glucose tolerance and salvaged pancreatic cells in diabetic rats. This combination has been suggested a new therapeutic approach for T2DM.

BCP present in *Copaifera duckei* extract has been attributed to attenuate hyperglycemia and hypercholesteremia in STZ-induced diabetic rats [55]. It also restored β-cells and increased number and diameter of the Langerhans islets as well as improved histology of pancreas. BCP presents as a major constituent in *Cinnamomum tamala* (Indian bay leaf or Indian cassia) has been screened in an in silico reverse screening approach to characterize the therapeutic targets of its components against targets involved in chronic disease treatment [56]. BCP was found to target calmodulin, heat shock protein 90-beta, cholinesterase and α-L-integrin. Further, in an in vivo study, BCP present in *Cinnamomum tamala* oil attributed to exhibit antidiabetic, antioxidant, and hypolipidemic activity in STZ-induced DM in rats [57].

In a non-controlled pilot clinical study, powdered dried leaves of *Eugenia punicifolia* has been given as adjuvant for three months in T2DM [21]. The extract significantly decreased glycosylated hemoglobin, basal insulin, C-reactive protein, and both systolic and diastolic blood pressure with no changes in fasting and postprandial glycemia. BCP present in the extract is attributed to its effects and suggestive of its potential as a candidate to be used in DM and may prevent complications.

Overall, these in vivo studies summarized in Table 2 depict that BCP administration in STZ-induced DM produced restoration of blood glucose and lipid levels, and increased insulin release. In addition, BCP treatment resulted in insulinotropic, anti-inflammatory and antioxidant properties, mitigating the altered activities of carbohydrate metabolic enzymes.

### 2.2. Effect on Insulin Resistance, Dyslipidemia, and Obesity

Zheng et al. [40] demonstrated a new role of the CB2R pathway by observations that it can enhance fatty acid oxidation rate and contribute to lipid homeostasis. Fatty acids are key functional and structural components of lipid metabolism and the abnormal oxidation of fatty acids showed to be associated with T2DM and obesity [61]. Rates of fatty acid oxidation are regulated at both transcriptional and non-transcriptional levels. At the transcriptional level, peroxisome proliferator-activated receptor-gamma coactivator 1a (PGC-1α) enhances the ability of the hormone nuclear receptors PPARα and ERRα to facilitate transcription of fatty acid oxidation enzymes [62]. The activity of PGC-1α is regulated by deacetylase Sirtuin1 (SIRT1) and SIRT1/PGC-1α pathway plays a key role in fatty acid oxidation [63]. Previous studies investigated that SIRT1 deacetylase activity is improved via different nutrient and signaling pathways, including glucose/calorie restriction and activation of AMPK and cAMP response element-binding protein (CREB) [64].

Among the three different isoforms, PPAR-α, mainly expressed in heart, liver, intestine, and macrophages is activated by polyunsaturated fatty acids and leukotrienes. Whereas PPAR-γ, mainly expressed in adipocytes is activated by polyunsaturated fatty acids and 15d-prostaglandin J2 and plays a vital role in adipocyte differentiation and lipid accumulation. Functionally, the PPAR isoforms resembles to those of the steroid receptors and linked to multiple functions initiated by nutrients, nutraceuticals, and phytochemicals. Ligand-binding to CB1R and CB2R enhances the activity of MAPK that further regulates the activation of PPARs through direct phosphorylation and PPAR activation provides a basis by which cannabinoids like BCP can regulate gene transcription triggered by dietary intervention. Several studies provide evidence that cannabinoids exert their anti-inflammatory properties, at least in part by activation of PPAR-α and PPAR-γ pathway [65,66].

Cannabinoids are known to interact or crosstalk with a family of peroxisome proliferated activator receptors (PPARs) including three distinct subtypes namely PPAR-α, PPAR-β/δ, and PPAR-γ that are encoded by distinct genes and are regulated by steroids and lipid metabolites and control lipid and glucose homeostasis and inflammatory responses [40,65,67,68]. The activation of CB2R on the surface of the cells triggers the intracellular signaling cascade that leads to the activation of PPARs [69]. The PPARs belongs to the members of nuclear receptor superfamily that functions as ligand-activated transcription factors and plays significantly in cellular proliferation, differentiation, organogenesis, inflammation, as well as the expression of hepatic enzymes regulating glucose homeostasis, insulin sensitivity, and lipid metabolism [70].

The activation of CB2R have shown to directly interact and activate PPAR-α and PPAR-γ [40,59,71]. BCP by activating CB2R triggers the activation of PPAR-α and PPAR-α signaling which exhibits several physiological and therapeutic effects in dyslipidemia, vascular inflammation, and IR [40,59]. It was found that BCP interacts directly with the ligand-binding domain of PPAR-α, employing several assays such as luciferase assay, surface plasmon resonance, and fluorescence resonance energy transfer assay [41]. BCP showed to regulate cellular lipid metabolism in a PPAR-α-dependent manner by reducing PPAR-α responsive gene expressions, intracellular triglyceride, uptake and oxidation of fatty acids. The equilibrium dissociation constants value of BCP for the PPAR-α was found 1.93 μM with an EC_50_ value of 3.2 μM [41]. The clinically used agonists of PPAR-α are fibrates that restore altered lipid profile and improves dyslipidemia and atherogenesis with the potential to reduce diabetic complications [72]. BCP has been shown to activate PPARγ, the main target of clinically used insulin sensitizers, thiazolidinedione drugs which are pharmacologically known as PPARγ agonists and used in IR and T2DM as insulin sensitizers [58]. Subsequent to CB2R activation, BCP showed to activate PPAR-γ in convincing number of studies and attributed to the therapeutic effects exerting anti-inflammatory and antioxidant effects.

In regards to its pharmacokinetic profile, BCP is highly lipophilic, possessing hydrophobic core in its structure, and a relatively small molecular weight of 204 g/moL compared with other conventional ligands targeting the PPAR isoforms. Moreover, the relatively small molecular weight, lipophilicity, and the absence of adverse effects, e.g., edema, osteoporosis, obesity, associated with synthetic ligands of PPARs are the advantages of BCP over conventional agonists of PPAR-γ which are clinically available for therapeutic management of DM. Additionally, BCP is a highly lipophilic compound, which renders it permeable enough to cross the cell membrane and showed its potential benefits and pharmacological actions by regulating the intracellular pathways involved in inflammation. However, the exact mechanism of this interaction remains elusive and this interaction needs to be explored for further exploration in therapeutic targeting.

Interestingly, BCP by activating CB2R as well as both of the PPAR isoforms (PPAR-α and PPAR-γ) offers a promising polypharmacological multitargeted approach for its pharmaceutical development and clinical application. Many studies demonstrated that dual agonist action on PPAR-α and PPAR-α are superior for metabolic control and dyslipidemia [68,72]. Considering the concerns and potential for the adverse effects with clinically available PPAR-γ agonists, BCP could be a safer alternative over synthetics being CB2R-selective agonist along with additional agonism on PPAR-α and PPAR-γ and being natural, non-toxicating, and devoid of the adverse effects of synthetics cannabinoids and PPAR activators. BCP may be developed to be used as an add-on or adjuvant with modern drugs to reduce their doses to avoid side effects and exert synergistic therapeutic effects.

**Figure 1:**
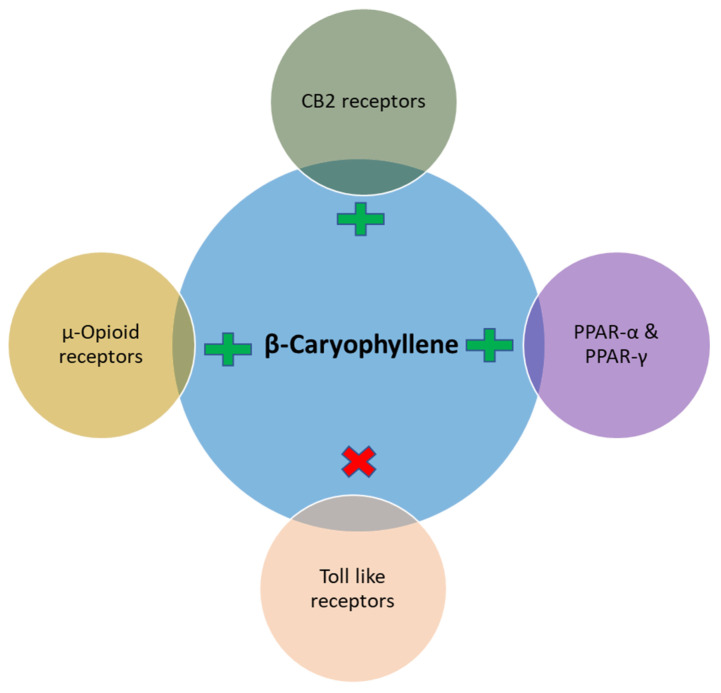
The receptor modulatory activity of β-caryophyllene.

Notably, in the majority of the experimental models involving chronic low grade inflammatory states similar to DM the principal pharmacological and molecular mechanism observed with BCP treatment is an improvement of immune dysregulation, inhibition of pro-inflammatory cytokines such as TNF-α, IL-1β, IL-6, NF-κβ, adhesion molecules and chemokines and subsequent modulation of signaling pathways mainly, toll-like receptors, opioid receptors, SIRT1/PGC-1α, AMPK/CREB, MAPK/ERK, Nrf2/Keap1/HO-1, and activation of nuclear peroxisome proliferator-activated receptors (PPARs, mainly PPAR-α, PPAR-γ) involving CB2R-dependent mechanism (Figure 1).

Further, BCP treatment to differentiated C2C12 myotubes stimulated SIRT1 deacetylase activity by increasing the phosphorylation of CREB, thus leading to enhanced deacetylation of PGC-1α [40]. BCP also increased the expression of genes, linked to the fatty acid oxidation pathway in a SIRT1/PGC-1a-dependent mechanism and hastened fatty acid oxidation rate in C2C12 myotubes, using siRNA to selectively knockdown CB2R results in the loss of BCP effect on the activation of SIRT1/PGC-1α pathway and fatty acids oxidation [40]. The results reveal that CB2R activation by a selective agonist induces lipid oxidation through a signaling/transcriptional pathway. Thus, CB2R- and SIRT1/PGC-1α-mediated mechanism of BCP may afford therapeutic options for metabolic diseases linked with lipid dysregulation [40].

A high-calorie diet is usually associated with obesity and IR, which predispose more diseases that are as serious as diabetes, cardiovascular diseases, and increase the risk of different neurological disorders. As claimed by various studies, diet-induced obese animals show higher anxiety and depressive-like behaviors [73]. Many mechanistic pathways are involved in neurobehavioral changes induced by diet including oxidative stress, inflammation, and IR [74]. Youssef et al. [59] have performed a study to evaluate the effect of BCP on high fat/fructose diet (HFFD)-induced metabolic and neurobehavioral changes in rats. The involvement of CB2R- and/or PPAR-γ-dependent activity was also investigated. BCP mitigated HFFD-induced IR, oxidative-stress, neuroinflammation, and behavioral changes. The anti-oxidant, anti-inflammatory, and anxiolytic effects of BCP were mediated by both PPAR-γ and CB2R activation. BCP was found to affect glycemic parameters mediating CB2R- and PPAR-γ-dependent mechanisms. Furthermore, antidepressant and memory improvement induced by BCP is mediated only via CB2R, largely by upregulation of PGC-1α and BDNF, a neurotrophin vital for neuronal growth, survival, repair and plasticity, cognitive function, and mood [75].

In another study, Youssef et al. [58] showed that BCP exerted protective effects against the negative consequences of dyslipidemia and vascular inflammation in mice. BCP remarkably inhibited the mediators involved both in inflammation as well as in atherosclerosis as TNF-α and NF-κB. BCP treatment resulted in VCAM1 suppression, a vascular cell adhesion protein, which promotes the adhesion of white cells of the vascular endothelium and favors atherosclerosis. Further, BCP normalized the ratio between eNOS and iNOS nitric oxide synthase within the aorta. iNOS is increased in atherosclerosis and in the phlogistic process following reactive oxygen species-induced NF-κB activation. Additionally, iNOS produces a high amount of nitric oxide, which interacts with reactive oxygen species, producing peroxynitrites, which exaggerate the oxidative stress. All these effects are attributed to a direct effect of BCP, as cannabinoid receptors CB2R agonist, other than of PPAR-γ receptors, involved in decreasing total cholesterol levels, low-density lipoprotein (LDL), and very low-density lipoprotein (VLDL), in the restoring of nitric oxide concentration and nitric oxide synthase isoforms and in the inhibition of vascular inflammation and synthesis of adhesion molecules.

In addition, it appears that BCP being PPAR-γ agonist leads to the reduction in fat mass and triglycerides and increases in high-density lipoprotein (HDL) [58]. These effects are mediated via the binding of BCP to CB2R, which activates PGC1-α and promotes the interaction between PPAR-γ and different transcriptional factors, such as PPAR-α leading to increase in the expression of enzymes involved in the oxidation of fatty acids in the liver. On comparison with pioglitazone, BCP was found more effective than the pioglitazone for all the parameters, except for the glutathione levels. Moreover, BCP did not cause body weight gain, the major adverse effect of pioglitazone [58]. Thus, BCP could be advantageous over conventional agonists of PPAR-γ which are clinically available for therapeutic management of DM.

The down-regulation of BDNF was implicated in the pathology of neurodegenerative diseases and many inflammatory conditions, such as acute coronary syndrome and T2DM [76]. It was concluded that BCP improves depression and memory deficit by modulating PGC-1α/BDNF pathway in a CB2R-dependent manner. Both CB2R and PPAR-γ are involved in anti-oxidant, anti-inflammatory, and anxiolytic effects of BCP. In addition, BCP improved depression and memory deficit by modulating PGC-1α/BDNF pathway in a CB2R-dependent manner. Overall, these studies summarized in Table 2 indicate that BCP administration resulted in increasing the rate of fatty acid oxidation and contribution to lipid homeostasis, an action mediated by SIRT1/PGC-1α and it regulated cellular lipid metabolism via PPAR-α activation. Therefore, BCP may provide therapeutic opportunities to treat metabolic diseases associated with lipid dysregulation.

### 2.3. Enhancement of Glucose Uptake in Tissues and Organs

Kumawat and Kaur [37] in an ex vivo study studied the effect of BCP in combination with L-arginine on the rate of glucose absorption across the everted sac and glucose uptake in rat diaphragm. Glucose utilization involves the transport of cellular glucose, which is mediated by glucose transporters (GLUTs). The GLUT4 subtype mainly present in skeletal muscles and adipose tissue carries solutes through a signaling pathway mediating insulin receptor tyrosine kinase [77]. Following stimulation with insulin, GLUT4 enhances the glucose uptake by translocating rapidly from cytoplasm to the plasma membrane. In DM, the impairment in the system of insulin-mediated glucose utilization may be due to defective GLUT4 translocation and aberrant insulin signal transduction [78]. The combination of BCP and L-arginine exerted remarkable glucose absorption in the small intestine and glucose uptake in the diaphragm. The combination had a pronounced effect on glucose absorption and uptake in tissues, however, the precise mechanism is yet to be elucidated.

Insulin signal transduction in normal physiological conditions involves complex sequential steps including many enzymes and mediators, resulting in facilitated glucose entry into the adipocytes, muscles, and myocardial cells via GLUT-4 (glucose transporter-4) transporters [79]. The insulin signal transduction is initiated by binding of insulin, insulin-like growth factor-1 (IGF-1), IGF-2 to the α chain of insulin receptors, which are members of the transmembrane tyrosine kinases receptors containing α and β chains [80]. This binding results in structural changes in the β chain by autophosphorylation of tyrosine residues and accompanied by downstream effects such as different adaptor proteins recruitment, i.e., insulin receptor substrates (IRSs), Shc (SHC-transforming) protein, and APS protein (adapter protein with a PH and SH2 domain) [81]. These processes allow IRS-1 (insulin receptor substrate-1) to have an appropriate binding site [82].

There are several types of insulin-dependent kinases such as ERK1/2 (extracellular signal-regulated kinase 1/2), PKC (protein kinase C), S6K1 (ribosomal protein S6 kinase beta-1), SIK2 (serine/- threonine-protein kinase 2), Akt (protein kinase B), mTOR (mammalian target of rapamycin), and ROCK1 (rho-associated protein kinase 1) and other types of kinases such as AMPK (AMP-activated protein kinase) and GSK-3 (glycogen synthase kinase 3) can phosphorylate and activate IRSs [83]. After that, activated IRS-1 binds to PI3K (phosphoinositide 3-kinase) stimulating its activation consequently catalyzes the conversion of PIP2 (phosphatidylinositol 4, 5-bisphosphate) to PIP3 (phosphatidylinositol 3,4,5-trisphosphate). PIP3 is a potent inducer for Akt, which in turn facilitates the entry of glucose into the cells by localization of GLUT-4 and by inhibiting glycogen synthase kinase leading to more glycogen synthesis [84].

BCP was also found to regulate the expression of several cytokines e.g., TNF-α, IL-1β, IFN-γ, IL-4, IL-5, IL-6, and IL-10, chemokines e.g., CINC-1/CXCL1, CXCL1/KC, MIP-2, MCP-1, growth factors e.g., vascular endothelial growth factor (VEGF), transforming growth factor (TGF), fibroblast growth factor (FGF), brain-derived neurotrophic factor (BDNF), enzymes e.g., acetylcholinesterase (AChE), butyrylcholinesterase (BChE), nitric oxide synthase (iNOS), NADPH oxidase (NOX), fatty acid transport protein (FATP4), ACC synthase (ACS), carnitine palmitoyltransferase 1 (CPT1), peroxisomal acyl-coenzyme A oxidase 1 (ACOX), cyclooxygenase-2 (COX-2), lipoxygenase (LOX), matrix metalloproteinase-9 (MMP9), myeloperoxidase (MPO), N-acetyl glutamate (NAG), mitogen-activated protein kinases (MAPK), signaling molecules (PGC-1α, PI3K/Akt, AMPK/CREB, ERK1/2, PKC, IGF, IRS1, S6K1, mTOR, TLR4, and GLUT4), adhesion molecules e.g., ICAM-1 and VCAM-1, apoptosis-related proteins e.g., Bcl-2, mdm2, cmyb, bax, bak1, Apaf-1, caspases, p53, p38, ATM, DR and Fas, proteins; cell cycle proteins e.g., cyclin D1, g-proteins e.g., Arf6, Rac1, Cdc42, heat shock proteins 60, and P-gP, genes e.g., Col1a1, Tgfb1, Timp1, SIR-2.1, SKN-1, DAF-16, mev-1, daf-16, SREBP-1c and SCD1 and receptors; CB2, μ-opioid, PPAR-α, PPAR-γ and α-nAchR. Additionally, BCP also alters the activity of several transcription factors and cell-signaling proteins (e.g., ERK, JNK1/2, NF-κB, AP-1, SIRT1 and STATs) and their signaling pathways [23]. The therapeutic potential of BCP in DM and its complications could be ascribed to favorable modulation of all these proteins and molecular targets which play critical role in the pathophysiology of DM by impairing insulin signal transduction and altered glucose homeostasis.

The metabolic effects of black pepper extract (PipeNig^®^-FL), containing high content of BCP have been assessed on glucose uptake and GLUT4 membrane translocation in C2C12 skeletal myotubes. PipeNig^®^-FL treatment to C2C12 myotubes stimulated cellular uptake of glucose and induced translocation of GLUT4. The effect of BCP rich extract in stimulating glucose uptake is due to improvement in GLUT4 trafficking to the plasma membrane. Consequently, GLUT4 storage vesicles translocation to the plasma membrane, largely in skeletal muscle and adipocyte, is directly associated with the ability to reduce raised blood glucose level [39].

Overall, these studies summarized in Table 1 demonstrate that BCP has potential to stimulate glucose uptake and absorption in skeletal muscle as well as adipose tissue mediating GLUT4 translocation.

### 2.4. Inhibition of α-Glucosidase Activity

α-Glucosidase (EC 3.2.1.20) in intestine are essential for carbohydrate digestion and involved in converting oligosaccharides and disaccharides to corresponding monosaccharides, which are then absorbed [85]. After a meal, hyperglycemia occurs in diabetic patients because of enhanced activities of α-glucosidase and α-amylase [86]. Therefore, inhibition of α-glucosidase prevents glucose absorption thus beneficial in reducing the blood glucose level after receiving carbohydrate diet [15,87]. Kaur G [38] determined antioxidant, anti-inflammatory, and α-glucosidase inhibitory activities of BCP, L-arginine and their combination in an in vitro study. BCP showed concentration-dependent inhibition of α-glucosidase activity and L-arginine was found more effective than BCP. However, the combination of BCP and L-arginine was superior to individual agents in inhibiting α-glucosidase.

Additionally, many plants such as Afromomum melegueta and Afromomum danielli [88], Phlomis armeniaca WILLD., Phlomis nissolii L., and Phlomis pungens WILLD. var. pungens [89], *Rhaponticum acaule* (L.) DC [90], Acacia mollissima and Acacia cyclops [91] and Neolitsea kedahense Gamble [92] and Senna podocarpa (Guill. and Perr.) [93] containing BCP as a major constituent have shown α-glucosidase inhibitory activity. The essential oils obtained from the seeds, flowers, leaves, and/or stems of these plants characterized to contain BCP as one of the major components and attributed to display potent free radical scavenging, xanthine oxidase inhibition, metal chelating, and antioxidant activities, in addition to inhibition of α-glucosidase [90]. These plants rich in BCP were suggested useful in T2DM, obesity, and hyperlipidemia in many studies [90].

Recently, the scaffolds of BCP and 4-hydroxycourmarin were used as a starting compound for the synthesis of novel scaffolds with better stability and improved bioactivities for α-glucosidase inhibitory activity [94]. Accordingly, various compounds have been synthesized using BCP [94]. The meroterpene-like derivatives synthesized from BCP and coumarins following biomimetic reaction exhibited potent inhibition of α-glucosidase. The natural meroterpenes derived from phloroglucinols and BCP showed high inhibitory activity against α-glucosidase. The stable conformers of caryophyllene favored the generation of enantioselective (4*R*,5*S*)-configuration products. The synthesized compounds were tested for their activity on *α*-glucosidase. Three derivatives with the molecular formula (C_25_H_30_ClO_3_, Molecular weight (M. wt.) = 413.1883), (C_25_H_38_O_3_Na, M. wt. = 409.2718), and (C_25_H_30_O_3_Na, M. wt. = 401.2092) formed potent inhibition of α-glucosidase and provided novel scaffolds for lead molecules in drug discovery for diabetes with promising inhibition (IC_50_ < 10 μM). The improvement inhibitory activities were due to the formation of a flexible connection between BCP and 4-hydroxycoumarin, and the presence of hydroxyl group which facilitates the binding. Linking rearranged BCP with aromatic ketones via an oxygen bridge generated compounds with better activity than acarbose, a standard drug. The presence of the *para*-substituted carbonyls and the hydrophobic moiety of BCP contributed to the formation of the compounds with higher inhibition [94].

In another study, the β-lactam motif of BCP following remodeling into different types of compounds and one of the derivatives showed potent inhibitory activity against α-glucosidase and it was found superior to the standard drug acarbose [95]. Further, in another study monoterpenoids-like derivatives were synthesized from BCP, as a starting compound and the derivatives containing caryophyllene moieties were recognized as a non-competitive inhibitor of α-glucosidase (IC50 < 15 μM), more potently than standard drug, acarbose [96].

Further, BCP has been attributed to exert inhibitory activity on pancreatic α-amylase, facilitates carbohydrate metabolism by interrupting α-1,4-glycosidic bonds to form oligosaccharides, that convert in to glucose [97]. Thus in addition to α-glucosidase, inhibiting α-amylase lessened hyperglycemia by delaying carbohydrate metabolism and reducing glucose absorption in intestine [97]. BCP present in many plants such as *Afromomum melegueta* and *Afromomum danielli* [88], *Phlomis armeniaca* WILLD., *Phlomis nissolii* L., and *Phlomis pungens* WILLD. var. *pungens* [89], and *Senna podocarpa* (Guill. and Perr.) [93] containing BCP as a major constituent has shown α-amylase inhibitory activity. The essential oil from methanol extract of *Citrus macroptera* Montr fruits containing BCP as one of the major ingredient showed α-amylase inhibitory activity and hypoglycemic activity in normal and glucose-induced hyperglycemic rats [60].

Pancreatic lipase, an enzyme, plays a significant role in the metabolism of dietary fat, thus pharmacological inhibitors of lipase have a therapeutic effect on obesity, protective effect in diabetes due to reduced absorption of lipids and restoration of insulin along with correction of lipid profile. BCP present in numerous plants such as *Ferulago nodosa* (L.) Boiss. [98], *Rhaponticum acaule* (L.) DC [90], *Artemisia annua* [99], *Ocimum kilimandscharicum* [100], *Pinus massoniana* L. [101] has been attributed to exert lipase inhibitory activity. One of the important lipase inhibitors used clinically in obesity and T2DM prevention is orlistat reported to display numerous side effects like alteration in liver enzymes, fecal urgency and incontinence, and steatorrhea. BCP being natural and safe on liver, stomach, and intestine in experimental and clinical could be a better alternative for therapeutic use [102]. However, further kinetic studies and preclinical and clinical trials are needed to establish its use.

The effect of BCP has been demonstrated on another enzyme, protein tyrosine phosphatase 1B (PTP1B) found in important insulin-targeted tissues such as liver, muscle, and fat and play a regulatory role in insulin signal transduction, by dephosphorylating activated insulin receptors or insulin receptor substrates. Excessive PTP1B activities impairs insulin sensitivity and signal transduction, thus participates in T2DM and regulate energy in brain mediating leptin receptors and contribute to obesity [97]. The inhibitors of PTP1B demonstrated to be useful in T2DM and obesity by favorable modulation of carbohydrate metabolism. One of the major concerns with the development of synthetic derivatives of PTP1B is the lesser cell permeability and selectivity [103]. BCP present in plants such as *Salvia amarissima* Ortega [104], *Blumea balsamifera* (L.) DC has been attributed to exhibit inhibitory activity PTP1B [105]. The high lipophilicity of BCP is advantageous for its cell permeability, thus could be a better alternative than synthetics.

**Figure 2:**
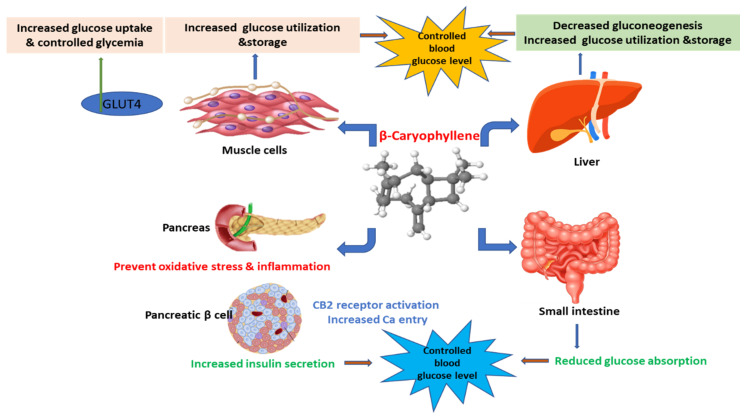
The mechanisms of β-caryophyllene on liver, muscle, pancreas, and small intestine to maintain normal blood glucose level. GLUT4; Glucose transporter type 4.

The available data indicate that BCP and its derivatives have the potential to inhibit the activity of α-glucosidase, α-amylase, lipase, and PTP1B that is suggestive of its underlying mechanisms of the usefulness of BCP in diabetes. BCP appears to be more potent than acarbose that further demonstrates its use as an adjuvant with other agents in T2DM and obesity. BCP and congeners could be developed as potent antihyperglycemic agents particularly to be useful in T2DM and obesity due to polypharmacological properties on fat and carbohydrate-metabolizing enzymes. The mechanism of the antihyperglycemic action of BCP involves increasing glucose utilization and uptake, affecting glucose metabolism, and increasing insulin secretion are represented in Figure 2. Though, further in vivo experiments are needed to confirm the in vitro observation in the efficacy.

## 3. β-Caryophyllene in Diabetic Complications

The complications of both T1DM and T2DM constitute retinopathy, nephropathy, neuropathy, cardiomyopathy, and other associated diseases which affect the quality of life and are a main reason for morbidity and mortality [24,26]. Diabetic complications arise from hyperglycemia associated with persistent oxidative stress and impairment in multiple metabolic pathways [106]. Chances of complications are inevitable despite treatment, therefore continued effort for the search of newer agents are ongoing for DM as well as its complications [107].

In several diseases involving immune dysregulation including obesity, DM and its complications, the activation of toll-like receptors (TLRs), particularly toll-like receptor 4 (TLR4), among many isoforms showed to garner great therapeutic interest, thus its pharmacological manipulation received interest in recent years [108]. TLR4 generates an active receptor complex that leads to the initiation of intracellular inflammatory signals and causes the onset and progression of inflammation in diabetic complications. In a very recent study, BCP was found to exert hepatoprotective effects mediating suppression of TLR4/receptor for advanced glycation end-products (RAGE) pathways [109] and neuroinflammation by attenuating TLR4/NF-*κ*B signaling pathway and inhibiting release of pro-inflammatory cytokines production and promotes polarization of M1/M2 phenotypic anti-inflammatory properties [110,111].

**Figure 3:**
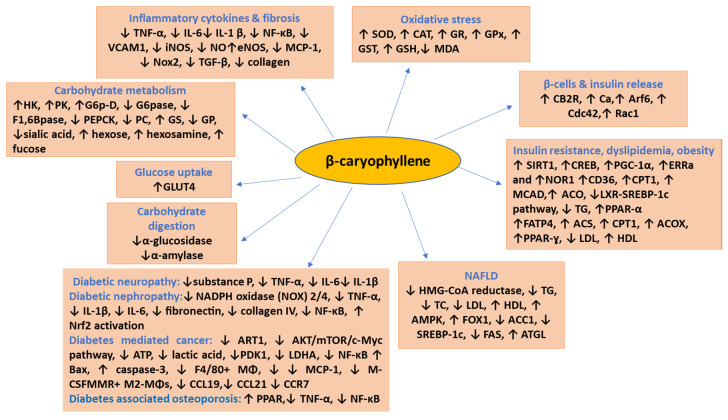
The molecular targets modulated by β-caryophyllene in diabetes and its complications.

Additionally, BCP has been shown to attenuate neuroinflammation and neuron death by mitigating high mobility group box 1 (HMGB1)-TLR4 signaling pathways [111]. HMGB1 following binding to TLR4 and RAGE results in the induction of inflammatory pathways via activation of NF-kB. TLR4 interfering PGC-1*α* enhances oxidative stress, inflammation, and cell death that leads to tissue damage in diabetic complications. The inhibitory effects of BCP on TLR4/RAGE, HMGB1-TLR4, NF-kB, PI3K/Akt, AMPK/CREB, ERK1/2/JNK1/2, SIRT1/PGC-1α, TLR4/NF-kB pathways are suggestive of its pharmacological and molecular mechanism of its potential usefulness in DM and its complications. The molecular targets of BCP are represented in Figure 3. In addition, BCP was found to significantly improve and augment endogenous antioxidants, scavenge free radicals and inhibit lipid peroxidation in blood and many organ tissues [29,58,59]. Therefore, BCP may have therapeutic potential in DM and its complications by pharmacological targeting of intimately linked oxidative stress-inflammatory cascade.

Neuropathic pain is considered a diabetes consequence and presents a high impact on patient’s quality of life, showing painful thermal, electrical, and sharp sensations [112,113]. Diabetic neuropathy is due to neuronal dysfunction, high excitability in the spinal horn, and lowered function of inhibitory neurons. Chronic hyperglycemia activates the polyol pathway and also promotes the release of advanced glycation end products, damaging nerve terminals, evoking pain, and increasing production of substance P and various cytokines such as TNF-α, IL-1β, and IL-6, which have a role in diabetic neuropathy development and permanence [114]. Aguilar-Ávila, et al. [115] demonstrated the effect of BCP on neuropathic pain and depressive-like behavior in experimental diabetic mice. BCP given orally to STZ-induced diabetic mice significantly reduced blood glucose levels and enhanced insulin levels. The decrease in blood glucose level is attributed to enhanced insulin secretion by BCP-mediating CB2R agonism. In addition, BCP treatment alleviated the pain associated with diabetic neuropathy. In addition, treatment with BCP significantly reduced substance P and inflammatory cytokines as neuropathic pain was mainly related to substance P and release of IL-6 and IL-1β.

A dietary supplement containing BCP, myrrh, carnosic acid, and PEA evaluated in twenty-five patients with the diagnosis of DM and painful distal symmetric polyneuropathy (PDSPN) showed a reduction of polyneuropathy with enhanced amplitude and reduced pain [116]. PDSPN is a common complication of DM involving perturbations of metabolic pathways regulating inflammation, microvessel circulation, and axonal degeneration. The formulation was found beneficial in painful diabetic distal symmetric sensory-motor neuropathy in patients with diabetes in an observational clinical study and tolerable with no side effects [116].

BCP role in diabetic nephropathy, a major cause of end-stage renal disease in diabetic patients involving structural and functional alteration in podocytes has been demonstrated using an in vitro model of high glucose-induced glomerular mesangial cells [117]. BCP treatment showed to inhibit cell proliferation, ROS production, and NADPH oxidase (NOX) 2/4 expression along with reduced pro-inflammatory cytokines viz., TNF-α, IL-1β, IL-6, NF-κB activation and increased Nrf2 activation. BCP also attenuated fibronectin (FN) and collagen IV (Col IV) in mesangial cells. The protective effects of BCP in diabetic neuropathy have been attributed to NF-κB and Nrf2 signaling pathway-based anti-inflammatory mechanism [117]. However, the effects observed in vitro need to be confirmed in the in vivo models of diabetic nephropathy.

Additionally, hyperglycemia a common manifestation in T2DM is also related with a higher risk of developing colorectal cancer evidenced by increased arginine-specific mono-ADP-ribosyltransferase 1 (ART1) levels in colorectal tissues from patients with T2DM compared to non-diabetic patients. The role of BCP on ART1 on glycolysis and energy metabolism has been shown in CT26 cells cultured and exposed to high-glucose conditions and in STZ-induced BALB/c mice transplanted CT26 tumor cell [118]. BCP treatment reduced the overexpression of ART1 in cells favorably modulated glycolysis and energy metabolism in CT26 cells by regulating the protein kinase B/mammalian target of rapamycin/c-Myc signaling pathway and glycolytic enzymes expression. BCP-induced reduction in the expression levels of ART1 via NF-κB indicates that ART1 is an attractive therapeutic target and BCP may be an attractive molecule for controlling cell proliferation, apoptosis, energy metabolism, tumor growth along with regulating metabolic pathways.

BCP showed potential in ageing and age-related disorders by improving the mean lifespan, reduced feeding behavior, induced dietary restriction like effects, and enhanced longevity-promoting stress resistance ability in *Caenorhabditis elegans* [119]. BCP also attenuated ROS accumulation and levels of intracellular lipofuscin, a marker of aging and age-related cellular damage in *Caenorhabditis elegans*. BCP enhanced the expression level of stress and longevity-promoting genes such as *daf-16*, *skn-1*, *sod-2*, *hsp-70*, *gst-4*, *gst-7*, *sir2.1*, *and sod-3*, the anti-oxidative enzymes induced in response to oxidative stress. Ageing as well as diabetes also enhances cognitive decline. BCP showed to enhance lifespan, memory performance in young as well as aged mice mediating potent anti-inflammatory properties on pro-inflammatory cytokine [120]. BCP has been identified as one of the adaptogens constituent in the *Kaempferia parviflora* rhizome extracts based on its positive effects on swimming tests of mice [121].

Along with ageing, diabetes and obesity are the risk factors for osteoporosis characterized by reduced bone mass through decreased osteoblastic osteogenesis and increased osteoclastic bone resorption. BCP was found to enhance osteoblastic mineralization and suppress adipogenesis and osteoclastogenesis in cultured mouse bone marrow cells [122]. Obesity and diabetes are also considered to increase the risk of cancer. BCP showed to suppress HFD-stimulated melanoma progression and lymph node metastasis in a high-fat diet (HFD; 60 kcal% fat)-induced melanoma progression in C57BL/6N mice [123]. Interestingly, BCP reduced the HFD-induced body weight gain, fasting blood glucose, solid tumor growth, metastasis of lymph nodes, proliferation of tumor cells, angiogenesis, and lymphangiogenesis. BCP also suppressed the number of lipid vacuoles and F4/80+ macrophage (MΦ) and macrophage mannose receptor (MMR)+ M2-MΦs in tumor tissues and adipose tissues surrounding the lymph nodes and reduced the CCL19 and CCL21 levels in the lymph node and CCR7 expression in the tumor. BCP also prevented lipid accumulation in the in vitro model of white adipocytes (3T3-L1), migration of monocytes, and secretion of MCP-1 in murine melanoma cells (B16F10), adipocytes and M2-MΦs, angiogenesis and lymphangiogenesis. The inhibition of accumulation of lipids, M2-cells, and CCL19/21-CCR7 axis partly demonstrate the underlying mechanism of BCP in inhibiting HFD-elicited development of melanoma under the conditions of high glucose levels.

In diabetic patients, increased incidence of impaired wound healing severely affects the quality of life leading to prolonged hospitalization and lower limb amputation [124]. Numerous factors such as age, obesity, malnutrition, and macrovascular and microvascular disease, contribute to wound infection and delayed wound healing in diabetic patients. The onset of hyperglycemia is one of the major reasons affecting cellular response to tissue injury, imbalance of wound healing by PMN leukocytes and fibroblasts, and delayed response to injury and impaired functioning of immune cells in DM. Wound healing involves re-epithelialization mediating growth factors, cytokines, and extracellular matrix components such as collagen, fibronectin, and elastin by keratinocyte, important cell of the epidermis, and proliferation of fibroblasts [124]. Koyama et al. [125] found that BCP promoted re-epithelialization and facilitated the healing of cutaneous wounds in mice. *Artemisia montana* Pampan essential oil containing BCP has been shown to promote skin regeneration in human keratinocytes and wound healing by increasing phosphorylation of Akt and ERK 1/2 and induced the synthesis of type IV collagen [126]. In another study, a hydrogel containing nano-emulsified BCP was developed and showed in vitro as well as in vivo wound healing activity [127].

BCP alone is an unstable compound as it oxidizes rapidly and is unable to permeate but the nano-emulsion formulation was found to exhibit improved stability for 60 days, superior bio adhesiveness and facilitated skin permeation than BCP alone. The nano-emulsion was found to exert a healing effect in the dorsal wound model by reducing lesions, oxidative stress, and inflammation comparable to a standard cutaneous healing agent (Dersani^®^ oil). Essential oils of many other plants such as *Eugenia dysenterica* DC leaves containing BCP was found to enhance in vitro skin cell migration and promoted angiogenesis with no cytotoxicity [128]. Similarly, BCP-rich essential oil of black pepper showed anti-proliferative activity by inhibiting Collagen I, Collagen III, and plasminogen activator inhibitor 1, important in inflammation and tissue remodeling for wound healing [129]. In another study, essential oil of *Aspilia africana* (Pers.) C.D. Adams rich in BCP showed wound healing ability by reducing wound bleeding, enhancing wound contraction, increasing concentration of basic fibroblast growth factor and platelet-derived growth factor along with increasing white and red blood cells [130]. These studies intend that BCP has the potential to be developed for dermatological use specially in wound healing.

The aforementioned studies summarized in Table 3 indicate that BCP might be a potential therapeutic agent for neuropathic pain, nephropathy, cognitive impairment, osteoporosis and delayed wound healing, and colorectal cancer developed in diabetic patients.

## 4. β-Caryophyllene in Nonalcoholic Fatty Liver Disease (NAFLD)

Hyperlipidemia, particularly hypercholesterolemia, is recognized as a major contributor to the fatty liver diseases, cardiovascular diseases, and carcinogenesis leading to health problems and death around the world [131]. A diet containing a large amount of fat is linked with higher risk of obesity and hyperlipidemia in preclinical models and humans as it increases levels of cholesterol and triglyceride (TG) in plasma and tissues [132]. NAFLD is considered as one of the chronic hepatic diseases, characterized by disproportionate accumulation of hepatic lipids in the absence of remarkable ethanol intake. NAFLD was found to range from general steatosis to severe nonalcoholic steatohepatitis (NASH), that may progresses to cirrhosis and hepatic cancer [133]. There is a direct relationship of steatohepatitis with metabolic syndrome that is identified by abdominal obesity, dyslipidemia, IR with or without hyperglycemia, and hypertension and studies have reported the relation of NASH with metabolic syndrome [134,135].

Sirichaiwetchakoon et al. [136] reported that *Pluchea indica* (L.), popularly known as Indian camphorweed used as a tea and health tonic in Southeast Asia was effective in mitigating hyperglycemia and dyslipidemia induced by high fat diet in mice. It showed to attenuate hyperglycemia and dyslipidemia by correcting altered lipid and hematological profile, restoring liver enzymes and renal function, and improving oral glucose tolerance along with histological salvage of kidney and liver. The benefits of this plant were ascribed to the high content of BCP and advocated to be dietary supplement/nutraceutical for hyperlipidemia, obesity, and T2DM.

Baldissera, et al. [137] investigated the role of BCP in hypercholesterolemia using a model of hyperlipidemia induced by Triton WR-1339 in rats, as well as its possible effect on hepatic antioxidant enzymes. Hyperlipidemic rats treated with BCP showed decreased total cholesterol, triglycerides, and LDL, similar to the reference drug simvastatin, while HDL levels did not increase following treatment. BCP treatment inhibited the activity of HMG-CoA reductase, as well as suppressed ROS and TBARS levels and improved antioxidant system. The study demonstrates that BCP exerts a hypolipidemic effect through suppression of the hepatic HMG-CoA reductase, like statins class of antihyperlipidemic drugs.

Harb, et al. [138] have examined the hypolipidemic effects of BCP in hypercholesterolemia induced by high cholesterol and fat diet in male Wistar rats. BCP significantly reduced serum total cholesterol, LDL cholesterol, and the atherogenic index and significantly increased HDL cholesterol level. Moreover, its alleviated liver injury is evidenced by reduced hepatomegaly, macrovesicular steatosis, and the activity of hepatic enzymes. Furthermore, it produced a significant increase in the activity of the antioxidant enzyme superoxide dismutase. BCP significantly suppressed free radical formation, inhibited the activity of hepatic HMG-CoA reductase, and subsequently inhibited endogenous cholesterol synthesis.

In recent years, AMPK activation appears promising as a therapeutic target for the prevention and treatment of obesity, DM, and steatosis [139]. AMPK, a serine/threonine kinase is involved in a variety of biological activities that maintain energy homeostasis by regulating lipids and glucose in liver [140]. The active AMPK phosphorylates and inactivates acetyl-CoA carboxylase 1 (ACC1), which is a rate-limiting enzyme in fatty acid synthesis [141]. In addition, AMPK modulates two critical transcription factors associated with homeostasis of lipids, i.e., sterol regulatory element-binding protein 1c (SREBP-1c), and forkhead box protein O1 (FoxO1). The AMPK-mediated phosphorylation of SREBP-1c precursor inhibits its cleavage, nuclear translocation, and transcriptional activity, results in decreased expression of fatty acid synthase (FAS) [142]. On the other hand, AMPK directly phosphorylates FoxO1 and then translocated to the nucleus, leading to transcriptional upregulation of adipose triglyceride lipase (ATGL) [143]. Kamikubo, et al. [144] evaluated hepatic lipid accumulation inhibitory activity in medicinal foods via involving AMPK using palmitate-overloaded HepG2 cells. The co-incubation of palmitate-exposed HepG2 cells with BCP resulted in a dose-dependent decrease in intracellular lipid content. Moreover, the palmitate-mediated lipid accumulation was significantly alleviated upon BCP pre-incubation. BCP prevented the translocation of SREBP-1c into the nucleus and FoxO1 into the cytoplasm through AMPK signaling, and therefore, induced remarkable downregulation of FAS and upregulation of ATGL, respectively. BCP produced activation of AMPK via CB2R-stimulated Ca^2+^ signaling pathway. The studies are suggestive of that BCP could be helpful in preventing and ameliorating fatty liver diseases including NAFLD.

Arizuka, et al. [145] have investigated BCP effects on the methionine- and choline-deficient diet-fed mice model of NASH with cardiometabolic diseases. In steatohepatitis, liver develops histopathological changes along with oxidative stress, inflammation, and fibrosis. In addition to improving liver function and salvaging liver tissues, BCP treatment showed to attenuate hepatic inflammation, oxidative stress, and fibrosis evidenced by reduced expression of cytokines (IL-1β, IL-6 and MCP-1), enhanced antioxidant enzymes (super oxide dismutase 2 and glutathione peroxidase 1) and increased NOX2 (reduced form of nicotinamide adenine dinucleotide phosphate (NADP) oxidase 2), and inhibited fibrotic markers (TGF-β and collagen), respectively.

Recently, BCP structure was utilized as a template for drug discovery [146]. Following the chemical modifications of BCP, several new bioactive compounds were synthesized which could make an impact in drug discovery targeting the other components of endocannabinoid system including activation of CB2R as well as the inhibition of the enzymes; FAAH, a the major endocannabinoid degrading enzyme and cyclooxygenase-2 (COX-2), an enzyme isoform participates in arachidonic acid metabolism and endocannabinoid signaling [22]. The anti-inflammatory drugs are well-0known to cause the gastrointestinal adverse effects including dyspepsia and peptic ulcer. However, the gastroprotective properties of BCP are an added advantage in encouraging it for anti-inflammatory properties without the appearance of gastrointestinal issues signaling [147]. Taken together, these observations point to the direction that BCP exerts its potent anti-inflammatory effect by multimodal mechanisms including the most important COX-2 enzyme, a target for newer anti-inflammatory drugs, coxibs.

The alcoholic extract of clove containing BCP showed to inhibit fatty acid synthase, an enzyme for de novo lipogenesis, inhibited weight gain, abdominal adipose tissue, lowered lipid accumulation in the liver by regulating the content of total triglyceride (TG), low-density lipoprotein cholesterol (LDL-C), and epididymal adipose tissue in HFD-induced obesity [148]. The extract was also found to inhibit the S-phase DNA replication of HepG2 cells and adipocyte differentiation of OP9 cells.

**Figure 4:**
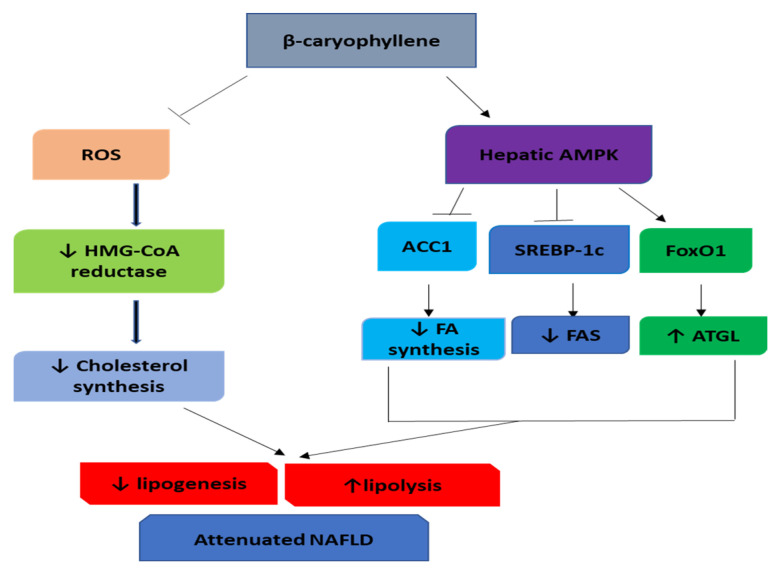
The molecular mechanisms of β-Caryophyllene in improving NAFLD. ROS, reactive oxygen species; AMPK, AMP-activated protein kinase; FA, fatty acid; FAS, fatty acid synthase; ATGL, adipose triglyceride lipase.

Altogether, these studies indicate that BCP has an antioxidant activity leading to the reduction of ROS, therefore, may be associated with deactivation of HMG-CoA reductase and decreased cholesterol synthesis, leading to hypolipidemia are represented in Figure 4. Also, BCP activated AMPK signaling via the mediation of the CB2R-dependent Ca^2+^ signaling pathway leading to the downregulation of FAS and upregulation of ATGL. Additionally, BCP could be a promising therapeutic drug in treating hypercholesterolemia and fatty liver disease.

## 5. Safety and Toxicity of β-Caryophyllene

BCP is classified as a substance of category five (toxic at doses greater than 2000 mg/kg) in accordance with OECD (Organization for Economic Co-operation and Development) guideline 423 [149]. BCP administered orally up to 2000 mg/kg did not elicit toxic effects in female Swiss mice [150] and the LD_50_ in rats were found greater than 5000 mg/kg [151]. BCP or its combination with L-arginine supplemented for 14 days at 2000 mg/kg was found non-toxic. Even BCP or the combination after repeated administration of 900 mg/kg did not show mortality [37]. Also, BCP did not show subchronic toxicity at the dose of 700 mg/kg/day in Wistar rats [151], and found safe on locomotion and muscle tone with both single and repeated doses in female Swiss mice [150]. In chronic good laboratory practices-compliant studies, BCP administered for duration of 90 days in Sprague-Dawley rats did not cause mortality or clinical toxicity. At high doses, it reduced body weight, food consumption, and efficiency due to palatability. Neither BCP nor epoxide derivative affected estrus cycle or sperms. Nephropathy and hepatocyte hypertrophy were observed at high doses. No observed adverse effect level (NOAEL) for BCP was found to be 222 mg/kg bw/day and for epoxide derivative was 109 mg/kg/bw/day [152].

In rodents, doses ranging from 20 to 300 mg/kg have been used to study pharmacological effects in drugs, xenobiotics or chemical toxicants-induced animal models of the heart [153,154], liver [109,155], kidney [156], colon [157], stomach [147], and brain [158,159] toxicity demonstrate its organoprotective properties and absence of the detrimental effect of these studied doses on organ structure and function [150,160]. Moreover, in various studies in female Swiss mice and in Wistar rats, no damage to the gastric mucosa and no changes in organs including brain, heart, liver, lungs, spleen, kidneys, or in hematology were observed [150,151]. In addition, Ames test has shown no mutagenicity [147]. However, bodyweight has reduced by 5% in female Swiss mice, but even if this variation was not significant [150].

BCP (20, 200, and 2000 mg/kg) was found antigenotoxic in benzo(a)pyrene-induced toxicity in mice by inhibiting the number of sister-chromatid exchanges and chromosomal aberrations [161]. The antigenotoxic activity was also shown to be related to its capacity to inhibit molecular oxidation and enhance glutathione-S-transferase (GST) activity. The effects of BCP obtained from curry leaf, a commonly used spice, on P-glycoprotein (P-gp) transport and CYP3A4 metabolism were evaluated in L-MDR1 (LLC-PK1 cells transfected with human MDR1 gene) and Caco-2 (human colon carcinoma) cells and CYP3A4 activity in pooled human liver microsomes [162]. The IC_50_ values of BCP on midazolam 1′-hydroxylation in HLM was 1.28 mM. This study indicated the potential of BCP drug interaction with other drugs metabolized by CYP3A4. BCP inhibited CYP3A activities in rats as well as in human hepatic microsomes rat and human hepatic subcellular fractions [163]. Therefore, BCP may cause drug-drug interaction with concurrently administered drugs. Recently in a placebo-controlled randomized double-blind trial for investigating efficacy in dyspepsia, BCP (126 mg/day) administered orally for eight weeks showed effective and tolerable. BCP being pleiotropic may synergistically provide greater therapeutic efficacy and safety by improving therapeutic outcome and minimizing risks/adverse effects compared to synthetic congeners. It has been found more potent than, probucol, tocopherol [164] and found synergistic with atorvastatin in reducing hematologic toxicity induced by chemotherapeutic agents [165]. Based on time tested safety due to dietary consumption and demonstrated efficacy in different animal models of diseases, BCP deserve further preclinical and clinical studies to promote clinical usage and pharmaceutical development. Though, in particular the long-term safety and toxicity of BCP still need to be evaluated.

## 6. Conclusions

Overall, the data from preclinical studies demonstrate the underlying mechanisms of BCP particularly in skeletal muscles, adipose tissues, liver, and pancreatic β-cells. The available studies demonstrate that BCP shows the capability to increase insulin secretion, insulin sensitivity, glucose uptake and reduce glucose absorption. In addition, it also reduces the levels of triglycerides and cholesterol, increases fatty acid oxidation and lipid homeostasis along with hypolipidemic effects. Moreover, BCP has insulinotropic effects mediating CB2R- and PPARs-dependent mechanism. BCP is one of the important components of essentials oils of many plants used in diet and traditional medicines for their well-being and health benefits. BCP is unique due to its selective full agonist property on CB2Rs as well as modulating other receptors and enzymes play significant role in glucose and lipid metabolism. However, further studies are required to translate these mechanistic studies into therapeutic benefits in humans.

Based on the health benefits, wide natural occurrence, dietary availability, low toxicity, relatively safe in humans use, and organoprotective properties with a plausible pharmacological affinity, selectivity and potency on different receptors and enzyme and molecular mechanisms targeting signaling pathways involved in glucose and lipid homeostasis, BCP appears to be a promising candidate for use in IR, T2DM, obesity, hyperlipidemia, and diabetic complications. The available literature reveals that BCP can be used as an adjuvant and reduce the doses of the currently used drugs and minimize their adverse effect and synergistically enhance therapeutic effects. Though, in perspective of pharmaceutical and nutraceutical development more pharmacokinetic and regulatory toxicity studies are required to ascertain the safety and efficacy for use in diabetes and associated complications.

## Figures and Tables

**Table 1:** Effects of β-caryophyllene: in vitro studies.

Cells	β-Caryophyllene Concentration/Duration	Effects	References
MIN6 β-cells	0.1–1 μM, 1 h	↑Insulin, ↑Arf6, Cdc42, and Rac1	[32]
Human Embryonic Kidney (HEK293)	20, 50, 100, 200 and 500 μM, 24 h	↓Oxidative stress, ↓Inflammation ↓α-glucosidase	[38]
Rat insulinoma (RIN-5F) cells	500 μmol, 24 h	↓Glucose absorption, ↑Glucose uptake, ↑Insulin secretion	[37]
C2C12 skeletal myotubes	BCP-enriched PipeNig^®^-FL extract 1, 10, 100 nM, 30 min	↑Glucose uptake, ↑GLUT4 translocation	[39]
C2C12 skeletal myotubes	1 μM, 48 h	↑p-SIRT1, ↑p-CREB, ↑Ac-PGC1α, ↑ERRa and ↑NOR1 (fatty acid oxidation transcriptional regulatory genes), ↑CD36 (fatty acid transport genes) ↑CPT1, MCAD, ACO (mitochondrial β-oxidation genes)	[40]
Lipid loaded HepG2 cells	1, 10 and 100 μM	↓LXR-SREBP-1c pathway, ↑PPAR-α ↓intracellular triglyceride, ↑FATP4, ACS, CPT1, ACOX	[41]

FATP4, fatty acid transport protein 4; ACS, acyl-CoA synthetase; CPT1, carnitine palmitoyl transferase; ACOX, acyl-CoA oxidase.

**Table 2:** Effects of β-caryophyllene on glycaemia: in vivo animal studies.

Animal	β-Caryophyllene Concentration/Duration	Blood Measures	Other Measures	Reference
Male Albino Wistar rats	200 mg/kg b.w., 45 days	↓Glucose, ↑Insulin, ↑Vit. C, ↑Vit. E, ↑GSH, ↑Ceruloplasmin, ↓MDA, ↓TNF-α and ↓IL-6 (in plasma)	↑Pancreatic SOD, CAT, GR, GPx, GST and GSH, ↓Pancreatic MDA, ↓Plasma TNF-α and IL-6	[29]
Male Albino Wistar rats	100 mg/kg, 200 mg/kg, 400 mg/kg, 45 days	↓Glucose levels ↑Insulin levels	↑liver, kidney and skeletal muscle HK, PK, G-6-PD, ↓liver, kidney and skeletal muscle gluconeogenic enzymes (G6pase, F1, 6Bpase, PEPCK and PC), ↑liver and skeletal muscle glycogen synthase, ↓liver and skeletal muscle glycogen phosphorylase	[42]
Male Wistar rats	200 mg/kg, 42 days	↓Glucose, ↓TGs, ↓SGPT, ↓SGOT ↓cholesterol	↑pancreatic GSH, ↑SOD, ↑catalase	[37]
Male Albino Wistar rats	200 mg/kg b.w., 45 days	↓Glucose, ↑Insulin, ↓hexose, ↓hexosamine, ↓fucose and ↓sialic acid	↓liver and renal sialic acid, ↑liver and renal protein-bound hexose, hexosamine and fucose	[46]
Male Wistar rats	30 mg/kg, P.O, 4 weeks	↓Glucose, ↓Insulin, ↓TC, ↓VLDL-c,ye ↓TG, ↓HOMA-IR, ↑HDL-c, ↓LDL-c	↑CB2-R, ↑PPAR-γ, ↑PPAR-α, ↑PGC1-α, ↓TNF-α, ↓NF-κB and ↓VCAM1, ↓MDA, ↑GSH, ↓iNOS, ↓NO, ↑eNOS	[58]
Male Wistar rats	30 mg/kg, P.O, 4 weeks	↓Glucose, ↓Insulin, ↓HOMA- IR	↑TAC, ↑GSH, ↓MDA, ↓NO, ↓TNF-α, ↓NF-κB, ↓iNOS, ↑PPAR-γ, ↑PGC-1α, ↑BDNF	[59]
Female Sprague Dawley rats	Ethanol extract of *Citrus macroptera* fruit (500 mg/kg and 1000 mg/kg, P.O)	↓Glucose levels at 2 h and 3 h after administration (hypoglycemic effect) ↓α-amylase	-	[60]
Male Wistar rats	*Copaifera duckei* containing BCP 21.25% (250 and 500 mg/kg, P.O, 30 days)	↓Glucose, ↓TG, ↓TC, ↓AST, ↓ALT, ↓urea and ↓creatinine	restore β-cells, ↑quantity and diameter of the Langerhans islets ↓liver mass	[55]

GSH, reduced glutathione; MDA, Malondialdehyde; SOD, superoxide dismutase; CAT, catalase; GR, glutathione reductase; GPx, glutathione peroxidase; GST, glutathione-S transferase; SGOT, serum glutamic-oxaloacetic transaminase; SGPT, serum glutamic pyruvic transaminase; HK, hexokinase; PK, Pyruvate kinase PEPCK, phosphoenolpyruvate carboxykinase; PC; pyruvate carboxylase; TGs, triglycerides; TC, total cholesterol; LDL-c, low-density lipoprotein-cholesterol; VLDL-c, very low-density lipoprotein- cholesterol; HDL-c, high-density lipoprotein-cholesterol; HOMA-IR, homeostatic model assessment of insulin resistance; NO, nitric oxide; BDNF, Brain-derived neurotrophic factor; ALT, alanine aminotransferase; AST, aspartate aminotransferase; BCP; β-caryophyllene.

**Table 3:** Effects of β-caryophyllene against diabetic complications.

Experimental Models	BCP Dose/Concentration/Period	Diabetic Complications	Effects and Mechanisms of BCP	References
Human mesangial cells	6.25, 12.5 and 25 μM for 1 h and then cells stimulated with high-glucose for 24 h	Diabetic nephropathy	▪ inhibited cell proliferation, ROS and NADPH oxidase (NOX) 2/4 expression ▪ lowered TNF-α, IL-1β, -6 levels ▪ suppressed fibronectin & collagen IV ▪ inhibited NF-κB and Nrf2 activation	[117]
STZ 40 mg/kg, i.p. at wk 1 (induction), STZ 120 mg/kg at wk 3, (reinforcement) to BALB/c female mice	10 mg/kg/60 μL, 45 days	Diabetic neuropathic pain	▪ reduced blood glucose and increased insulin levels ▪ alleviated diabetic neuropathy pain ▪ reduced substance P and pro-inflammatory cytokines	[115]
B16F10 melanoma cells-induced tumor and lymph node metastasis in high-fat diet (60 kcal%) C57BL/6N mice	0, 0.15 or 0.3% for 16 weeks with HFD	Diabetes associated cancer	▪ inhibited weight gain, tumor growth and metastasis ▪ inhibited cell cycle progression, cell survival, angiogenesis and lymphangiogenesis in tumors ▪ inhibited lipids and cytokine secretion	[123]
Mouse femoral tissues derived bone marrow cells	0.1–100 μM	Diabetes associated osteoporosis	▪ stimulated osteoblast mineralization ▪ inhibited adipogenesis by activating PPAR and osteoclastogenesis ▪ inhibited TNF-α and NF-κB	[122]
CT26 colorectal tumor cells exposed to high-glucose, and CT26 cells transplanted in STZ (100 mg/kg)-induced DM in male Balb/c mice	50 μM for 48 h in vitro and 200 mg/kg, P.O to mice for 10 days	Diabetes associated colorectal cancer	▪ reduced ART1 overexpression ▪ modulated glycolysis and energy metabolism ▪ upregulated protein kinase B/mTOR/c-Myc pathway & glycolytic enzymes expression ▪ suppressed proliferation and enhanced apoptosis of cancer cells	[118]
Distal symmetric polyneuropathy in patients with DM	Diet supplement containing BCP, myrrh, carnosic acid	Diabetic polyneuropathy	▪ beneficial in painful diabetic distal symmetric sensory-motor neuropathy ▪ tolerated with no side effects	[116]

STZ, Streptozotocin; HFD, high fat diet; DM, diabetes mellitus; ROS, reactive oxygen species; NADPH, nicotinamide adenine dinucleotide phosphate; PPAR, Peroxisome proliferator-activated receptor.
